# Research progress on debridement, antibiotics, and implant retention (DAIR) for the treatment of periprosthetic joint infection after artificial joint replacement

**DOI:** 10.1007/s00402-026-06368-3

**Published:** 2026-07-03

**Authors:** Wenguang Feng, Zhichao Zhang, Ribusurong Pu, Haibin Wang, Rui Liu, Yanhong Sun, Guoliang Zhang

**Affiliations:** 1 Department of Orthopedics, Affiliated Hospital of Inner Mongolia Medical University, Inner Mongolia Medical University, Hohhot, China; 2 Department of Emergency Surgery, Affiliated Hospital of Inner Mongolia Medical University, Inner Mongolia Medical University, Hohhot, China; 3 Inner Mongolia Medical University, Hohhot, China

**Keywords:** Debridement, antibiotics, and implant retention, Post-arthroplasty, Periprosthetic joint infection, Biofilm, Multimodal therapy

## Abstract

Periprosthetic joint infection (PJI) is one of the most serious complications following artificial joint replacement, posing significant treatment challenges and a heavy clinical burden. Debridement, antibiotics, and implant retention (DAIR) has become the preferred treatment strategy for acute PJI due to its advantages of minimal invasiveness, functional preservation, and cost-effectiveness. This article reviews the latest advances in optimizing the indications, timing of intervention, key technical details, and multimodal anti-infection strategies for DAIR. It focuses on discussing patient selection models (e.g., KLIC score), the impact of modular component exchange on biofilm eradication, the synergistic role of chemical debridement, and the central position of biofilm-active therapy in penetrating biofilms. Furthermore, this article proposes that future efforts should integrate molecular diagnostics, intelligent antimicrobial materials, and artificial intelligence prediction models to achieve individualized precision therapy for PJI, providing a theoretical basis for clinical decision-making.

## Introduction

Periprosthetic joint infection (PJI) is one of the most challenging complications following total hip and knee arthroplasty, with an overall incidence of approximately 1% to 2% [1]. It not only severely impacts patients’ quality of life but also imposes a significant burden on healthcare systems [2]. DAIR has become the preferred treatment strategy for acute postoperative PJI and acute hematogenous PJI due to its advantages of minimal invasiveness, functional preservation, and cost-effectiveness [3]. Since more than 2 decades, it is well known that by carefully choosing the patients for the DAIR-procedure according to an algorithm, the success rate is > 80% [4–6]. In recent years, with a deeper understanding of PJI pathogenesis, the criteria for patient selection, timing of intervention, and multimodal treatment strategies have been continuously optimized, leading to a more refined clinical application of DAIR [4–6]. This article aims to systematically evaluate the indications, influencing factors, and technical advancements of DAIR to improve treatment success rates and guide individualized therapy.

## Timing of debridement and implant retention surgery

Debridement and implant retention is a key strategy for treating PJI, particularly suitable for acute postoperative PJI (within 90 days of arthroplasty) and acute hematogenous PJI [7]. International consensus generally recommends performing DAIR when symptom duration is ≤ 4 weeks and the implant is stable, with interventions within 3 weeks of symptom onset associated with significantly higher success rates [8, 9].

Studies indicate that the efficacy of DAIR is closely related to “implant age” (i.e., the time interval from primary replacement to debridement). Intervention within 6 weeks postoperatively achieves infection eradication rates of 70% to 80% in reported studies, though these figures largely derive from selected patient cohorts or specialized referral centers with standardized protocols; real-world effectiveness may be lower and is considered the “golden window” for treatment [10]. In postoperative infections, a delay of DAIR beyond one month is associated with a significantly reduced success rate [11]. However, when the implant has been in place for more than 90 days, the success rate of DAIR decreases significantly, potentially related to mature biofilm formation, intensified local tissue reaction, and compromised fixation due to implant micromotion [12].

A multicenter study by Ottesen [13] et al. found that patients with an interval of more than 90 days from primary surgery to DAIR had a significantly increased risk of treatment failure, whereas intervention within 90 days achieved success rates as high as 90%. In contrast, for chronic PJI (typically exceeding a duration of 3–4 weeks), characterized by mature biofilm, high bacterial burden, and potential implant loosening, DAIR has limited efficacy. Systematic reviews show success rates of only about 50%, hence international guidelines typically do not recommend it [5].

DAIR should be considered a time-sensitive treatment, and its success depends on early diagnosis and timely intervention [5, 14, 15]. The ideal indications are PJI occurring within the first month after implantation, or presumed hematogenous PJI with a stable implant and a duration of symptoms of < 3 weeks. Implementing DAIR within this window can maximize functional implant retention, avoid complex revision surgery, and improve patient prognosis (Table 1) [16, 17].

## Patient-related factors in DAIR for PJI

DAIR is a key strategy for treating acute postoperative PJI and acute hematogenous PJI, and its efficacy is significantly influenced by various patient-related factors [16, 18]. Comprehensive preoperative evaluation and optimization of the patient’s systemic condition are essential for improving DAIR success rates and reducing recurrence risk [19].

### Impact of comorbidities

Obesity (BMI > 30 kg/m²) is an independent risk factor for PJI, and the infection risk increases significantly when BMI > 45 kg/m². Obesity not only increases surgical difficulty, leading to poor soft tissue perfusion, delayed wound healing, and insufficient antibiotic tissue penetration, but is also often accompanied by metabolic syndrome, further impairing immune function [20].

Diabetes mellitus significantly increases PJI risk due to microvascular complications and immunosuppression caused by chronic hyperglycemia. Preoperative glycemic control is crucial; a glycated hemoglobin (HbA1c) level < 7% is recommended to improve immune response and wound healing capacity [20, 21].

Immunosuppressed states (e.g., organ transplantation, chemotherapy, or long-term immunosuppressant use) also significantly increase infection susceptibility and treatment difficulty. Preoperative immune function assessment is required, with adjustment of immunosuppressive regimens when necessary, and consideration of immunoenhancing supportive therapy [20].

### Hepatic and renal function, and nutritional status

Hepatic and renal function directly affect the metabolism and excretion of antibiotics. Renal insufficiency may lead to drug accumulation, increasing toxicity risk. Abnormal liver function affects drug metabolism, necessitating individualized medication adjustments [22].

The KLIC score (Kidney, Liver, Index surgery, Cemented prosthesis, CRP value) has been proposed as a predictive tool for DAIR failure, as it comprehensively assesses renal function, liver function, type of surgery, use of cemented prosthesis, and C-reactive protein level. Patients with a score ≥ 4 have been reported to have a significantly increased failure rate (> 55%), suggesting that such patients may be more suitable for aggressive treatment strategies such as two-stage revision. However, the predictive value of the KLIC score remains controversial. Although some studies have validated its utility in predicting DAIR failure, external validation across different populations has yielded inconsistent results, with others suggesting limited applicability [23–25]. This highlights the need for further refinement and validation of the KLIC score in diverse clinical settings.

Nutritional status is closely related to immune function. Malnutrition leads to protein-energy deficiency, impairing immune function and tissue repair capacity, thereby increasing the risk of infection recurrence. Nutritional status should be systematically assessed preoperatively, and nutritional support rich in protein, vitamins, and trace elements should be provided to patients at nutritional risk [22, 24].

### Other reversible health issues

Uncorrected coagulation disorders predispose patients to postoperative hematomas, creating a “breeding ground for infection,” and must be screened for and managed preoperatively. Severe anemia affects tissue oxygen supply and immune cell function. It is recommended to correct hemoglobin to the normal range preoperatively, with iron supplementation or blood transfusion considered if necessary [26].

Furthermore, chronic underlying diseases such as cardiac insufficiency, chronic lung disease, and chronic kidney disease may all increase perioperative complication risks. Multidisciplinary collaboration is needed to optimize management and ensure the patient is in the best possible surgical condition [27].

The success of DAIR depends not only on surgical technique and microbiological management but also on the systematic assessment and optimization of the patient’s overall condition. By identifying high-risk factors, implementing preoperative risk stratification (e.g., KLIC score), and actively correcting reversible pathological conditions, the long-term efficacy of DAIR in treating PJI can be significantly improved [23–25, 28].

Among the multiple factors influencing DAIR outcomes, the timing of intervention (symptom duration ≤ 4 weeks, preferably within 90 days post-primary arthroplasty) and implant stability (absence of loosening) emerge as primary determinants of success. In contrast, patient-related factors such as obesity, diabetes, or nutritional status, while important, function mainly as secondary modifiers—they influence success rates by modulating host immunity and tissue healing but rarely reverse the indication for DAIR when the primary criteria are strictly met. This hierarchical understanding helps avoid overemphasis on modifiable comorbidities at the expense of delaying timely surgical debridement [27, 28].

## Key techniques and optimization strategies for DAIR

In the treatment of early periprosthetic joint infection after artificial joint replacement, the DAIR is indicated for acute PJI patients who meet strict selection criteria. However, its success or failure highly depends on the thoroughness of surgical debridement and the synergistic effect of multimodal intervention strategies [29].

### Exchange of modular components: a key step to enhance debridement efficacy

Whether modular components (e.g., polyethylene knee liners, acetabular liners, or femoral heads) should be exchanged during DAIR remains a focus of academic debate. Existing evidence suggests that exchanging modular components may significantly improve the success rate of infection eradication [30, 31].

Biomaterials research indicates that polyethylene surfaces are rougher than metal implants, making them more prone to bacterial adhesion and biofilm colonization, which makes it difficult to completely eradicate pathogens through mechanical debridement alone during surgery [30]. A systematic review by Tsang et al. [32] of cohort studies on DAIR for hip PJI from 1977 to 2015 found that the average success rate in the modular component exchange group was 73.9%, significantly higher than the 60.7% in the component retention group.

Bourget-Murray et al. [33] further confirmed in a prospective cohort study of 82 hip PJI patients that the treatment success rate with modular component exchange was 93.3%, compared to only 75.7% in the component retention group. Furthermore, a multicenter retrospective study including 349 hip and knee PJI patients caused by Staphylococcus aureus showed that exchanging modular components reduced the risk of treatment failure by 33%.

Nonetheless, some studies have found no significant impact of modular component exchange on DAIR success rates, suggesting its efficacy may be modulated by multiple factors such as pathogen type, infection duration, host immune status, and implant stability. Despite the controversy, the authors believe that, under the premise of ensuring implant stability, routinely exchanging biofilm-prone modular components such as polyethylene liners, acetabular liners, or femoral heads is an important means to achieve radical debridement, particularly suitable for early acute infections or PJI caused by low-virulence pathogens [34, 35].

### The role of chemical debridement and multimodal combination strategies

**Fig. 1:**
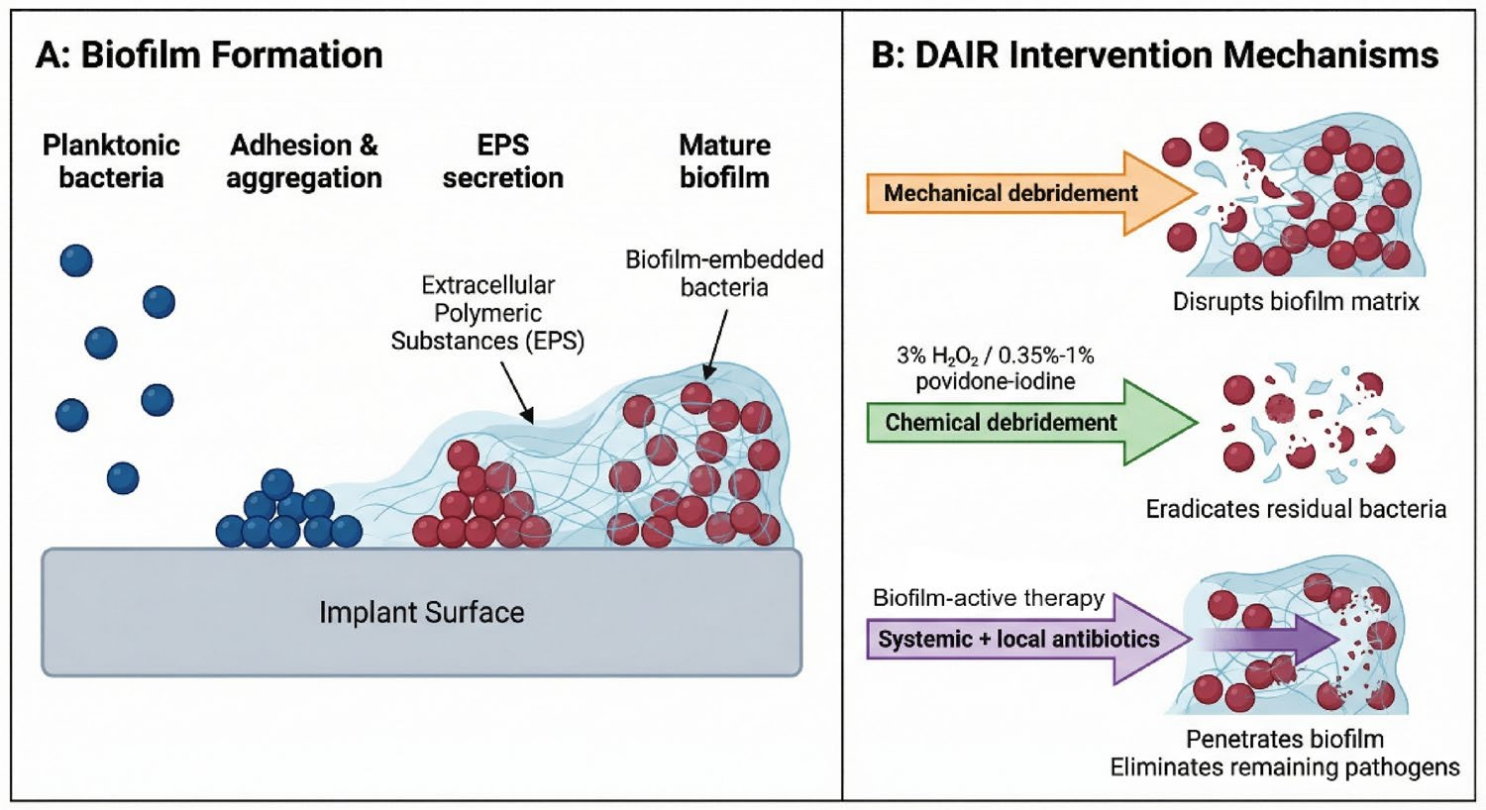
Schematic diagram of biofilm formation and DAIR treatment mechanism

Although mechanical debridement is fundamental for removing infected tissue, it is difficult to completely eliminate biofilms attached to the implant surface. Therefore, chemical debridement serves as an adjunctive measure, playing an irreplaceable role in reducing local bacterial burden and disrupting biofilm structure [36](Fig. 1).

Studies show that 3% hydrogen peroxide has good tissue safety and direct bactericidal ability, effectively inhibiting biofilm formation [37]. George et al. [38] reported that irrigation with 1% povidone-iodine combined with 3% hydrogen peroxide during one-stage revision achieved a PJI cure rate as high as 100%. Povidone-iodine has been incorporated into PJI treatment protocols by multiple international consensus statements.

A comparative analysis by Riesgo et al. [39] during DAIR procedures found that patients receiving chemical debridement with povidone-iodine had a treatment failure rate of 16.7%, significantly lower than those who did not (37%). Wan et al. [40] further noted that hydrogen peroxide and povidone-iodine individually have bacteriostatic effects, but their combined use exhibits synergistic bactericidal effects.

Based on evidence-based medicine, the 2018 International PJI Consensus recommends thorough irrigation with 6 to 9 L of fluid containing normal saline and bactericidal agents during debridement and implant retention surgery. The authors’ team routinely uses a staged irrigation protocol with normal saline, 3% hydrogen peroxide, and diluted povidone-iodine (e.g., 0.35% to 1%) to achieve dual mechanical and chemical debridement [41].

Notably, to avoid intraoperative cross-contamination, upon completion of debridement, the surgical team should routinely change gowns, gloves, and instruments to ensure subsequent procedures are conducted under “sterile” conditions. Although often overlooked, this step is crucial for reducing recontamination risk and improving the quality of debridement [23, 27, 39].

The success of DAIR surgery relies on the integration of multidimensional techniques: radical mechanical debridement, rational exchange of modular components, application of effective chemical irrigation solutions, and strict aseptic operative management. Future research should further elucidate the optimal combinations and sequences of various irrigation protocols and, combined with individualized assessment models, optimize the precision treatment pathways for PJI patients (Table 2) [39–41].

## The role of rational antibiotic selection, duration, and local application in DAIR for PJI

In the treatment of periprosthetic joint infection following artificial joint replacement, DAIR is the preferred treatment strategy for acute PJI, acute PJI. Its success depends not only on thorough surgical debridement but also on rational antibiotic selection, scientific treatment duration planning, and the organic integration of local and systemic therapy [42].

### Rational antibiotic selection and treatment Duration

Antibiotic selection should follow the principle of “empirical -> targeted -> de-escalation,” dynamically adjusted based on clinical features and microbiological evidence. Empirical antibiotic therapy plays a critical role in the management of PJI. However, to optimize microbiological yield, it should only be initiated after completion of debridement surgery and adequate collection of intraoperative tissue and synovial fluid specimens. Initiating antibiotics prior to sampling significantly reduces the sensitivity of culture and molecular diagnostics, potentially compromising the ability to identify the causative pathogen and tailor subsequent targeted therapy [43–45]. Once specimens are obtained, empirical therapy should be selected based on patient risk factors, infection timing, and local antimicrobial resistance patterns [43].

Typically, empirical regimens need to cover Gram-positive bacteria (especially Staphylococcus aureus and coagulase-negative staphylococci) and Gram-negative bacteria. For patients at high risk of resistance, initial therapy often includes vancomycin or daptomycin to cover methicillin-resistant Staphylococcus aureus. This is combined with cefepime, piperacillin/tazobactam, or a carbapenem to cover Gram-negative bacilli [44].

Once culture and susceptibility results from intraoperative tissue or synovial fluid are obtained, therapy should be rapidly switched to targeted treatment, selecting the narrowest-spectrum, highly sensitive antibiotic to reduce resistance risk and drug toxicity [45].

It is particularly noteworthy that rifampin holds an irreplaceable position in the treatment of staphylococcal PJI. Its efficacy is not solely attributed to biofilm penetration (a property shared by many antibiotics), but rather to its potent bactericidal activity against stationary-phase Gram-positive bacteria within the biofilm [47]. This unique mechanism allows it to effectively eradicate dormant pathogens adhering to implants. A randomized controlled trial by Zimmerli W et al. [46] confirmed that ciprofloxacin combined with rifampin achieved a success rate as high as 100% in treating orthopedic infections, significantly superior to monotherapy. Subsequent studies have also shown that rifampin combined with fluoroquinolones can significantly improve bacterial clearance rates and reduce recurrence risk. For PJI caused by Gram-negative bacilli, fluoroquinolones exhibit favorable biofilm penetration and bactericidal activity. Clinical studies have demonstrated that combination therapy significantly improves the infection eradication rate [47, 48].

### Mechanisms of DAIR failure and limitations of rifampin-based therapy

Failure of DAIR can be categorized into microbiological (persistent biofilm due to incomplete debridement or antibiotic-resistant small-colony variants), host-related (immunosuppression, poor nutritional status, uncontrolled diabetes), and mechanical (occult implant loosening or modular component exchange not performed). Among these, biofilm-mediated antibiotic tolerance remains the most recalcitrant [46].

Although rifampin-based combination therapy is considered the cornerstone for staphylococcal PJI, its use has notable limitations: (1) it is ineffective against Gram-negative bacilli; (2) rapid resistance develops if rifampin is used as monotherapy; (3) significant drug-drug interactions (e.g., with warfarin, direct oral anticoagulants, and immunosuppressants); and (4) poor tolerability (gastrointestinal, hepatotoxicity, and cytopenias) in up to 10–15% of patients. Therefore, rifampin should be prescribed only when susceptibility is confirmed, always in combination with a companion active antibiotic, and with close monitoring for adverse effects [42, 45, 47].

### Application of local antibiotics: a key strategy to overcome biofilm barrier

The core challenge in PJI lies in bacteria forming biofilms on the implant surface, leading to significantly increased tolerance to antibiotics. Therefore, the application of local antibiotics becomes an important means to compensate for the shortcomings of systemic therapy [49].

Local delivery methods are diverse, including antibiotic powder, solution irrigation, absorbable carriers, and non-absorbable carriers. PMMA antibiotic-loaded beads can achieve local high-concentration sustained release. Studies show success rates of 75% to 100% when combined with DAIR, but drawbacks include the need for secondary surgery for removal, uneven release, and potential to become new sites for bacterial colonization [45].

In contrast, the intraoperative use of antibiotic powder or solution irrigation offers advantages of simpler operation and lower cost. Local application of vancomycin powder can achieve synovial fluid concentrations far exceeding those from intravenous administration, maintaining effective concentrations for up to 24 h. Riesgo et al. placed 1 g of vancomycin powder in the fascial layer, increasing the DAIR success rate from 63% to 83.3% [50].

DAIR for PJI requires a synergistic “systemic + local” strategy. Rational antibiotic selection, adequate treatment duration, especially the application of rifampin-based combination regimens, combined with intraoperative local high-concentration antibiotic exposure, can effectively overcome the biofilm barrier and significantly improve treatment success rates [45, 48–50].

In addition to staphylococcal infections, DAIR outcomes may be compromised in cases of polymicrobial or fungal PJI, which are often associated with poorer prognosis and higher failure rates. Current evidence suggests that DAIR is less effective in such scenarios, and alternative strategies such as two-stage revision should be considered when these pathogens are identified preoperatively or intraoperatively [47, 50].

## Postoperative management of DAIR

### Follow-up and monitoring

Patients after DAIR require close follow-up to regularly assess infection control. Common monitoring indicators include inflammatory markers such as C-reactive protein and erythrocyte sedimentation rate. Imaging studies can also help assess implant stability and infection recurrence [51].

### Long-term antibiotic suppression

For some high-risk patients, long-term oral antibiotic suppression may be necessary to prevent infection recurrence. However, this is not standard practice and is typically reserved for individualized treatment in specific circumstances [51].

## Conclusion and future perspectives

DAIR, as a minimally invasive and cost-effective treatment method, shows promising prospects in the treatment of early PJI. However, its success depends on various factors including surgical timing, patient condition, surgical details, and antibiotic selection and usage. In contrast to DAIR, one-stage revision offers the advantage of a single procedure with high infection eradication rates (approximately 80–90%) in selected patients but requires extensive surgical expertise and is not suitable for all pathogens or bone defects. Two-stage revision, while associated with the highest reported cure rates (85–95%), imposes greater patient morbidity, longer hospitalization, higher cost, and functional loss during the interval period. DAIR remains the preferred strategy for early, stable implant infections, but when primary determinants (timing, stability) are unfavorable, one- or two-stage revision should be prioritized over repeated DAIR attempts.

Future research should continue to explore how to optimize DAIR treatment strategies, especially in complex cases, and develop new antimicrobial technologies and materials to improve treatment efficacy and patient quality of life. Novel antimicrobial materials such as nanocoatings and photodynamic therapy may bring new breakthroughs in PJI treatment. Simultaneously, personalized therapy will become a trend, involving the development of individualized treatment plans based on the specific circumstances of each patient to maximize therapeutic effects and reduce complications.

**Table 1:** Systematic summary of indications and contraindications for DAIR in treating PJI

Category	Recommendation	Specific Criteria
Ideal indications	Strongly recommended	Symptom duration ≤ 4 weeks, stable implant, no sinus tract, susceptible pathogen
Relative Indications	Consider with Caution	Symptoms 4–12 weeks, stable implant, low-virulence bacteria, no immunosuppression
Contraindications	Not Recommended	Chronic infection (> 3 months), implant loosening, multidrug-resistant bacteria, systemic sepsis

**Table 2:** Comparison and recommendations for irrigation solutions during DAIR

Type of irrigation solution	Mechanism of action	Recommended concentration	Notes
Normal Saline	Mechanical flushing, debris removal	4–6 L	Basic irrigation, no bactericidal effect
3% Hydrogen Peroxide	Physical biofilm disruption via bubble formation	Appropriate amount	Avoid intra-articular retention
Povidone-Iodine	Iodine ion bactericidal action, broad-spectrum antimicrobial	0.35%–1%	Requires final rinse to avoid soft tissue irritation
Antibiotic Solution	High local concentration bactericidal effect	Based on susceptibility	Requires combination with systemic therapy. Common antibiotics used for local irrigation include vancomycin (1–2 g/L) and tobramycin (1–2 g/L), often combined with normal saline. The choice should be guided by local antimicrobial susceptibility patterns and patient-specific factors.

## Data Availability

All data generated or analysed during this study are included in this published article [and its supplementary information files].
